# Implantable Defibrillators: Which Is the Best Device for Each Patient?

**DOI:** 10.1111/jce.16646

**Published:** 2025-04-02

**Authors:** Graham Peigh, Bradley P. Knight

**Affiliations:** ^1^ Northwestern University, Feinberg School of Medicine Chicago Illinois USA

**Keywords:** defibrillator, pacemaker, resynchronization

## Abstract

The first viable alternative to surgical implantation of an epicardial defibrillator system for secondary prevention of sudden cardiac death (SCD) was a single chamber transvenous implantable cardioverter defibrillator (ICD). Thanks to technological advancements over the past 40 years, electrophysiologists now have several options when determining the optimal type of ICD to provide protection against SCD, including the number of leads implanted, whether a patient meets indications for cardiac resynchronization, and if the patient would benefit from an extravascular device. In this review, we will detail the breadth of options for commercially available ICD therapy to provide guidance on which device is best suited for specific patient populations.

## Graphical abstract

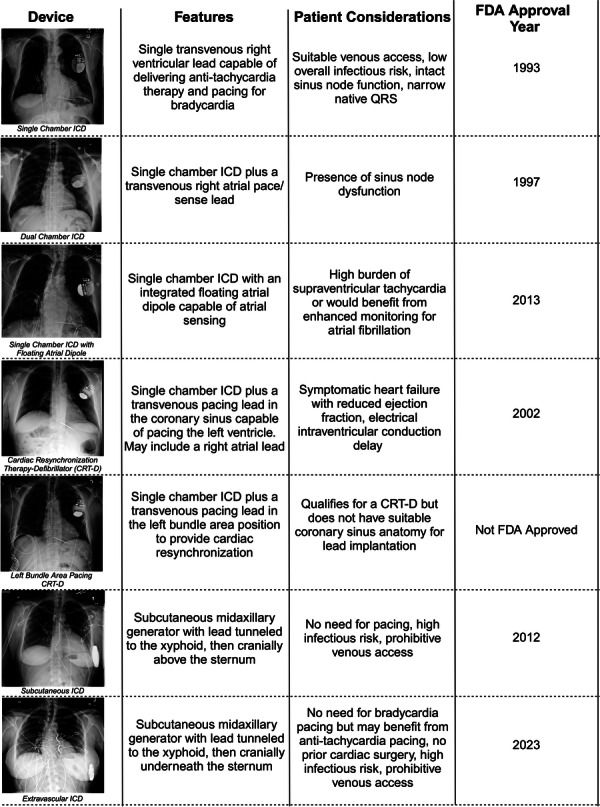

**Abbreviations**
AAD: antiarrhythmic drugs
AF: atrial fibrillation
CRT: cardiac resynchronization therapy
CRT‐D: cardiac resynchronization therapy‐defibrillator
DC: dual‐chamber
EV‐ICD: extravascular implantable cardioverter defibrillator
FDA: Food and Drug Administration
HF: heart failure
ICD: implantable cardioverter defibrillator
LBAD: left bundle area‐defibrillator
LBAP: left bundle area pacing
LVEF: left ventricular ejection fraction
NCDR: National Cardiovascular Data Registry
RV: right ventricle
SC: single‐chamber
SCD: sudden cardiac death
SVT: supraventricular tachycardia
S‐ICD: subcutaneous implantable cardioverter defibrillator
TV: transvenous
VF: ventricular fibrillation
VT: ventricular tachycardia

## 1 Introduction

Thanks to ongoing advancements in technology, electrophysiologists are now able to personalize the choice of which type of implantable cardioverter defibrillator (ICD) is implanted in a particular patient based on each patient's unique presentation, comorbidities, and preferences. The purpose of this review is to describe the range of ICD therapies that are available for primary and secondary prevention of sudden cardiac death (SCD) and outline the benefits and drawbacks of specific devices in various patient populations (Central Illustration 1).

**Central Illustration 1:**
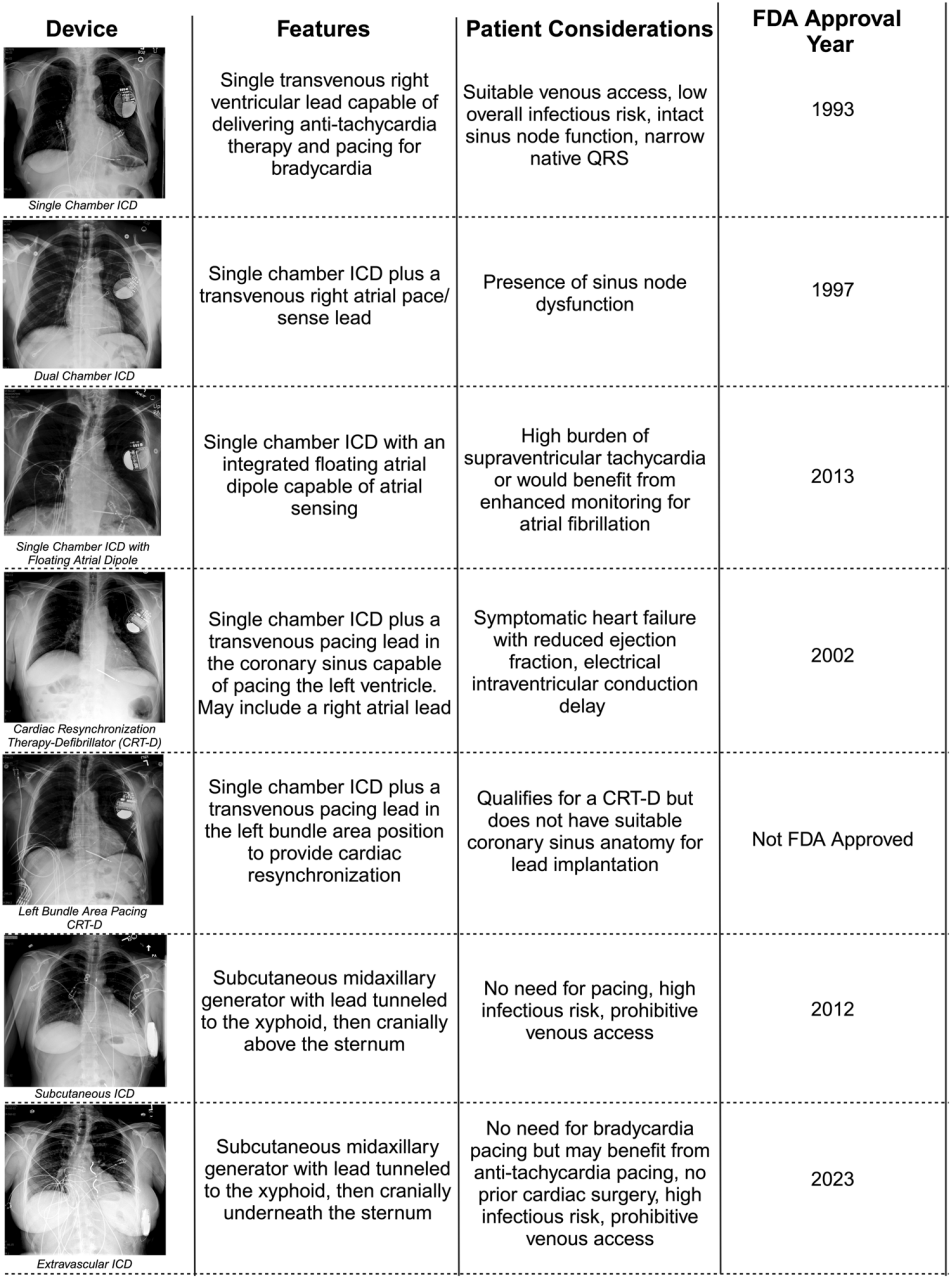
Which Implantable Defibrillator is Best for Each Patient? FDA, Food and Drug Administration; ICD, implantable cardioverter defibrillator.

## 2 Indications

### 2.1 Secondary Prevention Indications

The first implantable defibrillator was approved by the Food and Drug Administration (FDA) for secondary prevention of SCD in 1985 [1]. Following approval, several prospective trials compared mortality between SCD survivors who received ICD therapy and those who were prescribed antiarrhythmic drugs (AAD) for suppression of arrhythmia. Results from the antiarrhythmics versus implantable defibrillators (AVID) trial [2], Dutch Study [3], the Cardiac Arrest Study Hamburg (CASH) study [4], and the Canadian Implantable Defibrillator Study (CIDS) [5], all favored implantation of an ICD over use of AADs in SCD survivors, with a 20%–39% reduction in mortality associated with ICD implantation. Based on these results, ICDs now have a class I recommendation for secondary prevention of SCD in patients who survive SCD due to ventricular tachycardia (VT) or fibrillation (VF), those who have hemodynamically unstable VT, or those who have stable VT > 30 s if overall survival > 1 year is expected [6].

### 2.2 Primary Prevention Indications

Due to changes in the myocardial structural and electrical substrate, those with reduced, compared to preserved, left ventricular ejection fraction (LVEF) are at increased risk of SCD [7, 8]. Accordingly, numerous prospective studies have been conducted to determine the indications for, and utility of, primary prevention ICDs in patients with heart failure (HF).

Multicenter automated defibrillator implantation trial (MADIT) randomized patients with ischemic heart disease, LVEF < 35%, and inducible VT/VF to medical therapy or a primary prevention ICD. Results demonstrated a relative reduction in mortality among the ICD cohort in this high‐risk population [9]. The subsequent MADIT‐II trial also demonstrated a mortality benefit of ICD implantation, compared to medical, therapy in patients with a prior myocardial infarction, and LVEF < 30% [10]. Finally, SCD in heart failure trial (SCD‐HeFT) was a three‐arm trial comparing (1) optimal medical therapy to (2) medical therapy plus amiodarone and (3) medical therapy plus an ICD. Results demonstrated that ICD implantation was associated with a reduction in mortality compared to the two medical therapy‐only arms [11].

Based on these results, and others, ICD implantation is guideline supported for primary prevention of SCD in patients with ischemic or nonischemic cardiomyopathy, LVEF < 35%, and New York Heart Association Class II/III symptoms on optimal guideline directed medical therapy. In addition, primary prevention ICD implantation is recommended for patients with certain genetic cardiomyopathies including hypertrophic cardiomyopathy, arrhythmogenic right ventricular cardiomyopathy, certain channelopathies, left ventricular noncompaction, and idiopathic dilated cardiomyopathy [6].

It is important to note that the optimal HF medical therapy has evolved from that which was used in the seminal studies that demonstrated a mortality benefit of primary prevention ICDs. Indeed, contemporary optimal medical therapy, including sodium glucose cotransporter two‐inhibitors, angiotensin receptor neprilysin inhibitor, and glucagon‐like peptide‐1 receptor agonists are independently associated with reductions in HF‐associated mortality. Accordingly, future investigations that revisit the relative mortality benefit of ICDs in this contemporary era of guideline directed medical therapy are needed [12].

## 3 Choosing the Right ICD

### 3.1 Transvenous ICD

#### 3.1.1 Number of Transvenous Leads: One Lead Versus Two Leads

When a decision has been made to pursue TV‐ICD implantation, electrophysiologists must decide whether to implant a single chamber (SC) or dual chamber (DC) ICD. The primary reason to implant an additional pace/sense lead in the atrium is to provide atrial pacing in the setting of sinus node dysfunction [13]. Furthermore, due to a greater ability to discriminate supraventricular tachycardia (SVT) from VT, some studies have demonstrated a lower rate of inappropriate shocks with DC‐ICDs, compared to SC‐ICDs. Specifically, among 782 patients with a primary prevention ICD in a multicenter registry, DC‐ICDs were associated with a lower rate of inappropriate shocks compared to SC‐ICDs, despite the use of device‐specific VT discriminators [14]. However, thanks to continuous improvements in SVT discriminators, contemporary data on the relative rates of inappropriate shocks between SC and DC ICDs are mixed, as will be detailed below.

While often guided by the need for atrial pacing or presence of a concomitant atrial arrhythmia, DC‐ICDs are sometimes implanted at the discretion of the operator without a strong indication for an atrial lead. Indeed, among 266 182 patients in the US National Cardiovascular Data Registry (NCDR) ICD Registry 2010–2018 who did not have an indication for an atrial lead at the time of device implant, 49.3% underwent implantation of a DC‐ICD [15]. Although it may be tempting to add an atrial lead at the time of TV‐ICD implant “just in case” the patient develops an indication in the future, there are a number of considerations that favor implant of a SC‐ICD in the absence of an immediate indication for atrial pacing.

In a retrospective, propensity matched, cohort study of patients with an ICD and without an indication for pacing in the NCDR 2006–2009, presence of a DC‐ICD, compared to SC‐ICD, was associated with a greater rate of all‐cause device‐related complications [16]. Adding specificity to these results, a subsequent retrospective study of 15 940 patients who underwent primary prevention ICD implant 2015–2019 demonstrated that implantation of a DC‐ICD was associated with a greater rate of pneumothorax, hemothorax, and lead dislodgement in patients who did not have a pacing indication [17]. Subsequent international retrospective analyses have demonstrated similar results, with presence of a DC‐ICD being associated with a higher rate of reoperations than presence of an SC‐ICD [18].

Furthermore, while pacing and defibrillation leads are generally durable over decades, approximately 1%–3% of patients will require lead extraction for lead malfunction or endocarditis within 10 years of implantation [19]. This is relevant when deciding to implant a SC versus DC‐ICD as each additional TV lead necessitating extraction increases the risk of both procedural complications and mortality [19].

Finally, presence of concomitant SVT is often cited as a reason for implantation of a DC‐ICD, even when a patient does not have an indication for atrial pacing [15]. While early studies did demonstrate greater discrimination between SVT and VT among DC‐ICDs, contemporary waveform discriminators built into ICDs have allowed for SC‐ICDs to yield a very low rate of inappropriate anti‐tachycardia therapies. Indeed, numerous studies have demonstrated similar rates of inappropriate shocks in patients with DC‐ICDs and SC‐ICDs, when SVT discriminators are used [20, 21].

Taken together, the increased rates of device‐ and procedure‐related complications among patients with DC‐ICDs, combined with a similar incidence of inappropriate shocks between SC and DC‐ICDs, strongly discourage “prophylactic” implantation of an atrial lead in patients without an indication for atrial pacing.

#### 3.1.2 Number of Transvenous Leads: One Lead With Floating Atrial Dipole

To combine the potential benefits of atrial sensing without subjecting patients to the risks of implantation of an additional TV lead, Biotronik (Berlin, Germany) developed the integrated DX lead: a right ventricular (RV) ICD lead with an integrated floating atrial dipole capable of atrial sensing. Since approval, retrospective analyses of patients who underwent implantation of an ICD with a floating atrial dipole have demonstrated modest reductions in inappropriate shocks, compared to patients who underwent implantation of a traditional SC‐ICD [22]. To determine if use of an integrated atrial dipole reduces inappropriate shocks in a prospective manner, the SMART‐CONTROL study is a prospective, multi‐center, open‐label randomized trial currently enrolling patients undergoing implant of a SC‐ICD with floating atrial dipole, and randomizing the atrial dipole to sensing “on” or “off.” Patients will be followed for 2 years to determine any differences in rates of inappropriate anti‐tachycardia therapies [23]. The results of this prospective study will further clarify the utility of a floating atrial dipole in potentially reducing inappropriate therapies.

Furthermore, from a diagnostic perspective, atrial sensing may allow for detection of subclinical atrial fibrillation (AF) [24]. Indeed, among 150 patients, presence of a device with an integrated floating atrial dipole was associated with a higher rate of subclinical AF detection than patients with a traditional SC‐ICD, and similar rate of subclinical AF detection as patients with a traditional DC‐ICD [24]. In a separate retrospective study including 1841 patients with an integrated SC‐ICD with floating atrial dipole, independent adjudication of device‐detected atrial high‐rate events had a > 95% positive predictive value for true AF overall, and a 99.7% positive predictive value for AF duration of > 1 h [25]. In light of randomized trials showing a potential benefit of anticoagulation for device‐detected AF > 6 min, and numerous retrospective studies demonstrating associations between device‐detected AF burden, healthcare utilization, mortality, and stroke, addition of atrial sensing to a RV ICD lead may have unique diagnostic and therapeutic benefits in specific populations [26, 27, 28, 29].

This “VDD ICD lead” may therefore be a good option for those patients without sinus node dysfunction to provide VDD pacing in young patients with AV block, patients at high risk for future AV block, or for patients with hypertrophic cardiomyopathy with obstruction who might benefit from pacing to reduce their left ventricular gradient.

#### 3.1.3 Number of Transvenous Leads: Three Leads

Patients with left ventricular systolic dysfunction who are candidates for ICD implantation often have biventricular delay in electrical conduction [30, 31, 32]. Cardiac resynchronization therapy (CRT), through pacing the left ventricle via a coronary sinus branch, attempts to restore biventricular synchrony, and therefore improve myocardial efficiency, through timed electrical activation of the right and left ventricles. Inclusion of a right ventricular defibrillation lead (CRT‐D) also provides protection against SCD [33].

Numerous trials have demonstrated the benefit of CRT‐Ds in select populations. Specifically, the Comparison of Medical Therapy, Pacing and Defibrillation in Heart Failure (COMPANION) trial randomized patients with severe symptomatic HF, LVEF ≤ 35% and presence of interventricular conduction delay (QRS > 120 ms) to CRT vs medical therapy. Results demonstrated that CRT was associated with a significant reduction in subsequent HF hospitalization and death [34].

Subsequently, the Multicenter Automatic Defibrillator Implantation Trial – Cardiac‐Resynchronization Therapy (MADIT‐CRT) compared outcomes of minimally symptomatic HF patients implanted with a traditional ICD to those implanted with a CRT‐D. Among 1820 patients with LVEF ≤ 30%, QRS ≥ 130 ms and NYHA I‐II failure symptoms randomized to receive a CRT‐D or traditional ICD, implantation of a CRT‐D lead to a significant reduction in the endpoints of death or HF events [35]. Importantly, all patients in this trial had uniform device programming, thereby setting the standard for contemporary brady‐ and anti‐tachycardia programming among patients undergoing CRT‐D implantation [36].

Finally, Resynchronization‐Defibrillation for Ambulatory Heart Failure Trial (RAFT) evaluated outcomes of mild‐moderate symptomatic HF patients implanted with a traditional ICD to those implanted with a CRT‐D. Among 1798 patients with NYHA II–III symptoms, LVEF ≤ 30%, and native QRS ≥ 120 ms or paced QRS ≥ 200 ms randomized to receive an RV apical ICD or a CRT‐D, there was a significant reduction in death and HF events among the CRT‐D cohort [37].

Taken together, the results from these three seminal CRT‐D studies showed a benefit of CRT compared to medical therapy in patients with a wide native QRS, depressed LVEF, and NYHA I‐III symptoms. Indeed, CRT is supported by a class I recommendation for patients with LVEF < 35%, a left bundle branch block with QRS duration > 150 ms and NYHA II‐IV (ambulatory) symptoms on optimal medical therapy to reduce mortality, decrease hospitalizations and improve quality of life. Furthermore, CRT is supported by a class II recommendation for patients with LVEF < 30%, a left bundle branch block with QRS duration > 150 ms, and NYHA class I symptoms on optimal medical therapy to reduce risk of worsening HF symptoms [38].

Achieving traditional CRT via inserting a left ventricular pacing lead into a coronary sinus branch may be limited by individual coronary sinus anatomy (suboptimal branch location for resynchronization), difficulty cannulating the coronary sinus, diaphragmatic stimulation and elevated pacing thresholds [39, 40]. Recently, there has been growing interest in CRT through direct stimulation of the conduction system using a pacing lead (conduction system pacing). Conduction system pacing may be achieved via a lead implanted in the His bundle or the left bundle. His bundle pacing was the first widely utilized method of conduction system pacing. While effective, His leads were impacted by increased thresholds due to insulation around the His bundle, leading to premature generator battery depletion [38, 41]. Accordingly, the most common contemporary variety of conduction system pacing for CRT is left bundle area pacing (LBAP).

In cases of LBAP‐CRT, the LBAP lead is used as the primary pacing lead with the RV ICD lead programmed to be offset in the QRS, or to a subthreshold output. Numerous retrospective studies have investigated the outcomes of LBAP‐CRT to traditional CRT. A metanalysis of 12 such studies demonstrated that LBAP‐CRT was associated with a relative increase in LVEF, improved symptoms, shorter QRS duration and decrease in HF hospitalizations compared to traditional CRT [42]. Despite promising retrospective results, the data on CRT achieved by LBAP remain limited compared to that supporting traditional CRT. Accordingly, while LBAP‐CRT may be used as a backup in the case of failed traditional CRT, future prospective head‐to‐head randomized trials are necessary for LBAP‐CRT to be guideline recommended as a first‐line strategy for cardiac resynchronization [38].

Finally, individual case studies have reported implantation of a defibrillator lead in the left bundle position to utilize a single lead for both LBAP‐CRT and defibrillation (LBAD). This would reduce the number of intracardiac leads from three to two. In a single center case series of five patients who underwent attempted LBAD lead implantation, two had left bundle capture and successful defibrillation threshold testing [43]. Significantly more data on the safety and efficacy of LBAD, particularly with regard to lead stability, feasibility of extraction, and defibrillation threshold trends, are needed before routine implementation.

### 3.2 Extravascular ICD

While usually durable, the long‐term presence of TV pacing or defibrillation leads is not without risk. Specifically, in a retrospective analysis of 20 580 patients with TV ICDs followed for 2.3 ± 2.1 years, the rates of lead‐related and infectious complications were 5.3% and 1.9%, respectively [44]. Additional consequences associated with TV ICDs include lead‐related tricuspid regurgitation and vascular occlusion [45, 46, 47, 48, 49]. Lead‐related complications often necessitate lead extraction or revision—both of which are associated with their own procedural risks [19]. Furthermore, some patients who necessitate an ICD have prohibitive venous access as a result of prior procedures. As a result, numerous extravascular ICD systems have been developed.

#### 3.2.1 Subcutaneous ICD (S‐ICD)

The S‐ICD system consists of a generator implanted along the left 5th/6th mid‐axillary line, with a shock lead tunneled from the generator to the xyphoid process and then over the sternum. This design provides three distinct shock vectors capable of delivering defibrillation for patients with an indication for an ICD, and no indication for pacing.

Safety and efficacy of the S‐ICD was established in a cohort of 314 patients, of whom 13% had a previous TV‐ICD. Through 6 months after implantation, 99% of patients remained free of device‐related complications, and the conversion rate from VF was > 90%. However, 13.1% of patients in this cohort experienced an inappropriate shock [50]. Since then, additional discriminators and algorithms have been added to the S‐ICD to improve the specificity for VT detection and decrease inappropriate shocks [51, 52]. Specifically, among 876 patients in the PRAETORIAN trial who were randomized to receive a TV‐ or S‐ICD, there was no difference in the rate of inappropriate shocks delivered by an S‐ICD compared to a TV‐ICD [52]. Importantly, device‐replated complications through 4 years of follow‐up were lower in the S‐ICD cohort (1.4% vs. 6.6%; *p* = 0.001) [52].

Subsequent evaluations of the S‐ICD, including in the UNTOUCHED trial, demonstrated similarly encouraging results. Among 1116 patients who underwent implant of an S‐ICD, the rate of inappropriate shocks was 4.1%, the successful conversion rate from VT or VF was 98.4%, and 92.7% of patients remained free from device‐related complications through 18 months of follow‐up [53].

Finally, in a retrospective analysis of 16 063 patients in the NCDR registry, there were no differences in adjusted all‐cause mortality, device reoperation, need for device removal, cardiovascular readmission or all‐cause readmissions through 2000 days of follow‐up between patients who underwent implant of a TV‐ versus S‐ICD [54].

Currently, implantation of the S‐ICD is supported by a class I indication in patients who qualify for an ICD, do not need pacing, and do not have adequate vascular access or do have a very high infectious risk. Furthermore, S‐ICD implantation is supported by a class II recommendation in patients who qualify for an ICD and do not have a current or anticipated need for bradycardia or anti‐tachycardia pacing.

### 3.3 Developments in S‐ICD Implantation Technique

Since its original description, there have been significant developments in S‐ICD implantation technique which have allowed for a broader range of patients to be good candidates for S‐ICD implantation. Originally, S‐ICD implantation necessitated three incisions: one in the mid axillary line for the generator, one over the xyphoid process for a suture sleeve, and one over the cranial aspect of the sternum to anchor the distal aspect of the lead. The two‐incision technique for S‐ICD implantation eliminates the incision over the cranial aspect of the sternum and has been associated with similar outcomes as the three‐incision technique [55]. By eliminating an incision, the procedure is less invasive, and the result may be more cosmetically pleasing for the patient.

Initially, an S‐ICD test shock was necessary to obtain system impedance after device implantation, with impedance < 90 ohms being associated with successful defibrillation. As a result of updates in the S‐ICD software, physicians are now provided with a low voltage system impedance via a sub‐threshold 1 V impulse. A multicenter retrospective analysis demonstrated strong correlation between high and low voltage impedance measurements. In some cases, a reassuring low voltage impedance may obviate the need for defibrillation threshold testing, thereby lowering the overall risk profile of the implantation procedure and minimizing time under anesthesia [56].

Finally, there was early concern about the efficacy of the S‐ICD in obese patients due to increased amounts of subcutaneous fat between the device and the chest wall increasing shock impedances. However, by modifying the location of the generator from above the latissimus dorsi muscle to between the latissimus dorsi and the serratus anterior, the amount of subcutaneous fat between system components and the chest wall is limited. This intramuscular generator implantation is associated with a high rate of successful defibrillation across the spectrum of body mass index [57].

### 3.4 S‐ICD Limitations

Despite these developments, certain limitations of the S‐ICD remain. Approximately 8% of patients who may benefit from the S‐ICD may not qualify due to T wave oversensing by the device [58]. Furthermore, the S‐ICD battery is prone to premature depletion [59]. Importantly, the S‐ICD is not capable of pacing [60]. Therefore, careful consideration needs to be given before S‐ICD implantation to anticipate a potential need for pacing. Indeed, in a multi‐center analysis, 4/22 patients who underwent S‐ICD extraction over 4 years required a device capable of pacing [61]. To address this shortcoming, the MODULAR study recently investigated the short‐term outcomes associated with a leadless pacemaker that communicated with a S‐ICD to provide bradycardia and anti‐tachycardia pacing. Results demonstrated a low rate of procedural complications and high rate of device‐device communication through 6 months of follow‐up, however long‐term outcomes remain unknown [62].

#### 3.4.1 Extravascular ICD (EV‐ICD)

The EV‐ICD consists of a TV‐ICD‐sized generator implanted in the 5th/6th midaxillary line, with a lead tunneled from the generator to the xyphoid process and then cranially underneath the sternum. Due to its substernal position, the EV‐ICD can provide both anti‐tachycardia pacing and defibrillation. The EV‐ICD is not able to pace for bradycardia and cannot be implanted in patients with prior cardiac surgery.

The safety and efficacy of the EV‐ICD was established in a prospective study of 316 patients who underwent device implantation. Within this cohort, 98.7% of patients had successful defibrillation threshold testing < 30 J at the time of device implant, and the rate of procedural or device related complications was 7.4%. In addition, the 1‐year rate of inappropriate shocks from the EV‐ICD was 10.2%, mostly due to cardiac oversensing. As expected, the rate of inappropriate shocks due to cardiac oversensing decreased over the study duration. Importantly, shocks were avoided in nearly half of all spontaneous episodes of ventricular tachycardia due to the availability and success of anti‐tachycardia pacing [51, 63]. While there are individual case reports of combining the EV‐ICD with a leadless pacemaker, there are no prospective data on the safety and efficacy of this technique [64].

Based on these results, the EV‐ICD is a viable alternative for patents without prior cardiac surgery who necessitate an ICD and do not have pacing indications, but may benefit from anti‐tachycardia pacing. Recommendations on the EV‐ICD have not yet been incorporated into major society guidelines.

### 3.5 EV‐ICD Limitations

Despite favorable data surrounding the efficacy of the EV‐ICD, some limitations do exist. In particular, due to the positioning of the substernal coil, P wave oversensing can lead to inappropriate oversensing of tachycardia. Indeed, in the EV‐ICD pivotal trial, there were 34 inappropriate shocks for P wave oversensing, accounting for 42% of the overall inappropriate shocks experienced [63]. Since then, there have been algorithms developed to detect p‐wave oversensing by assessing differential amplitudes of P and R waves, which may decrease inappropriate shocks by > 20% [65]. Additional methods to reduce P wave oversensing by the EV‐ICD include placement of the electrode slightly left of the midsternal line and avoidance of ring1‐ring2 sensing [66]. Despite these modifications, P wave oversensing remains a concern with the EV‐ICD, and future studies are needed to determine further risk factors and mitigation strategies for inappropriate shocks.

## 4 Unique Patient Populations

Due to the unique aspects of commercially available ICDs, certain devices may be particularly well suited for specific patient populations. For example, in patients who have high risk of infection and do not require pacing, the S‐ICD or EV‐ICD are appealing options [67]. Furthermore, extravascular ICDs are oftentimes favored in young patients, particularly those with genetic cardiomyopathies, given the risks of long‐term transvenous lead placement [68]. An exception to this general principle is in patients with hypertrophic obstructive cardiomyopathy who may benefit from forced right ventricular pacing to relieve left ventricular outflow tract obstruction [69].

Given the appealing nature of extravascular ICDs in young patients, who are likely to participate in sport, prior studies have demonstrated the safety and efficacy of S‐ICDs in athletes. Specifically, within a cohort of 1493 patients in a S‐ICD registry, there was no difference in rates of device‐complications or inappropriate shocks between those who did, and did not, regularly participate in athletics [70].

While S‐ICD, rather than TV‐ICD, implantation may be favored in patients on dialysis given the prevalence of difficult or prohibitive vascular access in this cohort, prior analyses have demonstrated that S‐ICDs have a higher rate of inappropriate shocks in dialysis patients, compared to patients not on dialysis [71]. Furthermore, in a retrospective analysis of 1371 dialysis patients who underwent ICD implantation, 39% of whom had a S‐ICD implanted, there was no difference in mortality, device complications, or hospitalizations based on the type of ICD implanted.

Taken together, while there are general principles that may favor a specific type of ICD implant for various groups of patients, the ultimate decision regarding the particular type of device to implant always depends on individual patient characteristics, preferences, and the operator's knowledge of implant techniques.

## 5 Conclusion

In this review, we have detailed the range of commercially available ICDs available for patients who necessitate primary or secondary prevention from SCD. In general, a TV SC‐ICD remains the mainstay of ICD therapy by allowing for anti‐tachycardia therapy and bradycardia pacing through a single TV RV lead. In patients who necessitate atrial pacing, a TV DC‐ICD is a strong alterative, though there are short‐ and long‐term risks to implanting a right atrial lead. For HF patients with significant interventricular conduction delay, a CRT‐D may improve long‐term HF outcomes. While a LBAP pacing lead is an attractive alternative to a traditional CS lead for CRT, future head‐to‐head prospective studies are needed to determine the outcomes of this technique. Finally, for patients who do not require pacing, extravascular ICDs provide protection from SCD without subjecting patients to the risks of TV leads (Central Illustration 1).

Despite these overarching guidelines, the decision of “which ICD is right for which patient” comes down to the patient presentation, preferences, and operator experience. Fortunately, with ongoing advancements in ICD technology, electrophysiologists may continually personalize the selection of implanted devices to suit each patient's unique needs.

## Disclosure

Dr. Knight receives speaker honoraria and consulting fees for Abbott, Biotronik, Boston Scientific, and Medtronic.

## Data Availability

The authors have nothing to report.
